# Estimated cerebral oxyhemoglobin as a useful indicator of neuroprotection in patients with post-cardiac arrest syndrome: a prospective, multicenter observational study

**DOI:** 10.1186/s13054-014-0500-6

**Published:** 2014-08-29

**Authors:** Kei Hayashida, Kei Nishiyama, Masaru Suzuki, Takayuki Abe, Tomohiko Orita, Noritoshi Ito, Shingo Hori, J-POP Registry Investigators

**Affiliations:** Department of Emergency and Critical Care Medicine, School of Medicine, Keio University, 35 Shinanomachi, Shinjuku-ku Tokyo, 160-8582 Japan; Department of Primary Care and Emergency Medicine, Kyoto University Graduate School of Medicine, Kyoto, Japan; Keio University School of Medicine, Department of Preventive Medicine and Public Health, Center for Clinical Research, Minato Tokyo, Japan; Division of Emergency and Critical Care Medicine, Saiseikai Yokohamashi Tobu Hospital, Yokohama, Japan; Department of Cardiovascular Medicine, Kawasaki Saiwai Hospital, Kawasaki, Japan

## Abstract

**Introduction:**

Little is known about oxyhemoglobin (oxy-Hb) levels in the cerebral tissue during the development of anoxic and ischemic brain injury. We hypothesized that the estimated cerebral oxy-Hb level, a product of Hb and regional cerebral oxygen saturation (rSO_2_)_,_ determined at hospital arrival may reflect the level of neuroprotection in patients with post-cardiac arrest syndrome (PCAS).

**Methods:**

The Japan Prediction of neurological Outcomes in patients with Post cardiac arrest (J-POP) registry is a prospective, multicenter, cohort study to test whether rSO_2_ predicts neurologic outcomes after out-of-hospital cardiac arrest (OHCA). This study assessed a subgroup of consecutive patients who fulfilled the J-POP registry criteria and successfully achieved return of spontaneous circulation (ROSC) from OHCA. The primary outcome measure was the neurologic status at 90 days.

**Results:**

We analyzed data from 495 consecutive comatose survivors who were successfully resuscitated from OHCA, including 119 comatose patients with prehospital return of spontaneous circulation (ROSC; 24.0%) and 376 cardiac arrests at hospital arrival. In total, 75 patients (15.1%) presented with good neurologic outcomes. Univariate analysis revealed that the cerebral oxy-Hb levels were significantly higher in patients with good outcomes. Multivariate logistic regression using the backward-elimination method confirmed that the oxy-Hb level was a significant predictor of good neurologic outcomes (adjusted odds ratio, 1.27; 95% confidence interval (CI), 1.11 to 1.46). Analysis of the area under the receiver operating characteristic curve (AUC) revealed that an oxy-Hb cut-off of 5.5 provided optimal sensitivity and specificity for predicting good neurologic outcomes (AUC, 0.87; 95% CI, 0.83 to 0.91; sensitivity, 77.3%; specificity, 85.6%). The oxy-Hb level appeared to be an excellent prognostic indicator with significant advantages over rSO_2_ and base excess, according to AUC analysis. The significant trend for good neurologic outcomes was consistent, even in the subgroup of patients who achieved return of spontaneous circulation on hospital arrival (1^st^ quartile, 0; 2^nd^ quartile, 16.7%; 3^rd^ quartile, 29.4%; 4^th^ quartile, 53.3%; *P* < 0.05).

**Conclusions:**

The cerebral oxy-Hb level may predict neurologic outcomes and is a simple and excellent indicator of neuroprotection in patients with PCAS.

**Trial registration:**

UMIN Clinical Trials Registry UMIN000005065. Registered 1 April 2011.

## Introduction

Although neurologic sequelae are common among survivors of out-of-hospital cardiac arrest (OHCA), no early prognostic markers have been reliably established [1-4]. The main objective of neurologic assessment of survivors with post–cardiac arrest syndrome (PCAS) in the acute postresuscitation period is not only to determine the ongoing injury but also to establish the patient’s recovery from unresponsiveness [3]. Because the brain is highly susceptible to ischemia, global cerebral ischemia often results in neurologic impairment after OHCA, regardless of whether a return of spontaneous circulation (ROSC) occurs [5,6].

Immediate high-quality cardiopulmonary resuscitation (CPR) is crucial for optimal patient outcomes [7,8]. The purpose of CPR is to provide effective oxygenation to the vital organs, particularly the brain and heart, through the artificial circulation of oxyhemoglobin (oxy-Hb) until ROSC is achieved [7]. The intended effect is to stop the processes of ischemia/anoxia caused by inadequate circulation and oxygenation [9]. The restoration of blood perfusion to the cerebral tissue and the capacity for oxygen delivery are strongly associated with anoxic brain damage during and after cardiac arrest. Notably, oxy-Hb levels and cardiac output are essential determinants of oxygen delivery during ongoing CPR attempts. However, little is known about oxy-Hb levels in the cerebral tissue during the development of anoxic and ischemic brain injury.

Recently, cerebral oximetry with near-infrared spectroscopy (NIRS) has been developed as a noninvasive technology that may be used for monitoring cerebral oxygen saturation during cardiac arrest [10-12]. Regional cerebral oxygen saturation (rSO_2_) can be continually measured by using the Beer–Lambert law [12,13], which describes the ratio [oxy-Hb/(oxy-Hb + deoxyhemoglobin)] × 100 [14]. The estimated cerebral oxy-Hb level, which is the product of blood hemoglobin (Hb) and rSO_2_, is described as [Hb (g/dl) × rSO_2_ (%)]/100, and it can reflect the cerebral oxy-Hb level during and after resuscitation.

We hypothesized that the estimated cerebral oxy-Hb level obtained on hospital arrival may reflect the level of neuroprotection in patients who are successfully resuscitated after OHCA. This study aimed to determine whether the estimated cerebral oxy-Hb level is a simple and effective predictor of 90-day neurologic outcomes in patients with PCAS.

## Materials and methods

### Study design and settings

The Japan Prediction of neurological Outcomes in patients with Post cardiac arrest (J-POP) registry was a prospective multicenter cohort study that was conducted from May 15, 2011, to August 30, 2013, and involved 15 tertiary emergency hospitals.

### Patient selection

Patients included in the J-POP registry were unresponsive during resuscitation on hospital arrival after OHCA. To collect maximum clinical data from the real-world setting, we included both comatose patients with detectable pulses and those with sustained cardiac arrest at arrival. Exclusion criteria were as follows: (a) trauma, (b) accidental hypothermia, (3) age younger than 18 years, (d) prior completion of a “Do Not Attempt Resuscitation” form, and (e) a Glasgow Coma Scale score of >8 on hospital arrival. This study assessed a subgroup of consecutive patients with OHCA who fulfilled the J-POP registry criteria. In addition, we excluded those who were declared dead in the emergency room, those not admitted to the hospital despite receiving advanced life support consistent with current CPR guidelines, and those with Hb levels of <7.0 g/dl. We collected only “post–cardiac arrest status” data for the present study.

### Data collection

Data were prospectively collected with an Utstein-style data form that included the following: age, sex, bystander–witness status, and presence and type of bystander CPR; origin of cardiac arrest, location of cardiac arrest, and initial documented cardiac rhythm; advanced airway management, use of epinephrine, or defibrillation by emergency medical service (EMS) personnel; time interval from EMS call to hospital arrival and ROSC before hospital arrival; administration of coronary angiography, primary percutaneous coronary intervention, or therapeutic hypothermia as a treatment after hospital admission; and arterial pH, base excess, Hb levels, and rSO_2_ on hospital arrival. Arterial pH, base excess, and Hb levels were obtained within 10 minutes of hospital arrival. Data management was performed by an independent data-coordinating center (Kyoto University Graduate School of Medicine, Kyoto, Japan).

### EMS in Japan

Emergency services are provided 24 hours every day, and the free emergency telephone number “119” is used to call for an ambulance from anywhere in Japan. Most ambulance crews have three EMS personnel, including at least one emergency life-saving technician with extensive emergency care training. Emergency life-saving technicians can insert adjunct airways, intravenous lines, and can use semiautomated external defibrillators during OHCA. All EMS personnel perform CPR according to the 2010 Japanese CPR guidelines [9,15]. EMS personnel are not permitted to terminate resuscitation in the field; therefore, most OHCA patients are transported to hospitals [16].

### NIRS

Immediately after hospital arrival, aiming within 3 minutes, two disposable NIRS probes (INVOS 5100C; Covidien, Boulder, CO, USA) were applied carefully and bilaterally onto the patient’s forehead. rSO_2_ was stabilized over a period of several seconds. The values were monitored for at least 1 minute, and the lower of the two measured rSO_2_ values was adopted for analysis. The cerebral oximeter emits 2 wavelengths of near-infrared rays (730 nm and 805 nm) into the patient’s forehead: it calculates spatial depth resolution by subtracting the shallow from the deep measurement, minimizes superficial signal contamination from the scalp and skull, and detects changes in Hb saturation in the brain [17,18]. The limits of detection for Hb–oxygen saturation were <15% or >95% on the basis of a cortical tissue depth of >2 cm [12]. This device interprets a reading of 15% as equivalent to no detectable cortical oxygen [12].

### Study end points

The neurologic outcome was determined on the basis of assessments at 90 days after hospital admission. The cerebral performance category (CPC) score was used to categorize neurologic outcomes as follows: CPC 1, good performance; CPC 2, moderate disability; CPC 3, severe disability; CPC 4, comatose or persistent vegetative state; and CPC 5, brain death or death. Good neurologic recovery was defined as CPC 1 and 2. CPC scores were further dichotomized into good outcomes (CPC 1 or 2) and poor outcomes (CPC 3, 4, or 5), which is an accepted categorization when evaluating patients with PCAS. The primary end point was good neurologic outcomes at 90 days after cardiac arrest [3].

### Data analysis

Baseline characteristics were summarized in the two groups defined by the primary end point (good or poor neurologic outcomes). Multivariate logistic regression with the backward-elimination method was used for adjusting multiple selected covariates to assess the association between potential predictors and outcomes. Candidate variables were predefined by clinical importance [19] and from the results of univariate analyses for the primary end point. In general, approximately 10 or more events per variable were accepted for multivariate modeling [20]. To evaluate the potential predictors as prognostic tools, receiver operating characteristic (ROC) curve analyses were performed for evaluating the accuracy in differentiating between good and poor neurologic outcomes at 90 days. Nonparametric estimates of the area under the ROC curves (AUCs) and their 95% confidence intervals (CIs) were calculated.

### Statistical analysis

Continuous variables were presented as mean ± standard deviation (SD) or median (25^th^ or 75^th^ percentiles), depending on the distribution of the variables, and were compared by using the Student *t* test (or analysis of variance) or Mann–Whitney *U* test. Categoric variables were presented as frequencies with percentages and were compared by using the chi-square test or the Fisher Exact test. Bonferroni correction was used to adjust for multiplicity. Multiple logistic regression using the backward elimination method was used to assess factors associated with 90-day good neurologic outcome. The Hosmer–Lemeshow test was used to assess the goodness-of-fit of multiple logistic regression models. AUCs between two pairs of potential predictors were compared by using a nonparametric test [21]. The linear trend in a proportion across a factor was tested by means of the exact Cochran–Armitage trend test. Significance levels for all tests were two-sided and were set at *P* < 0.05. All data were analyzed with SPSS version 19.0 (SPSS Inc., Chicago, IL, USA) and SAS version 9.2 (SAS, Cary, NC, USA).

### Ethical considerations

Each participating center’s Institutional Review Board (IRB) reviewed and approved the study (see Acknowledgements) and waived the requirement for written informed consent according to the relevant ethical guidelines for observational research [22].

## Results

### General characteristics

**Figure 1:**
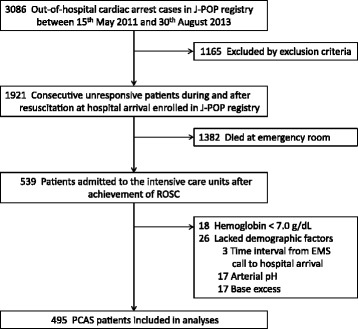
**Patient selection.**

In total, 3,086 adult patients with OHCA were referred to the participating emergency hospitals, 1,921 of which were consecutively enrolled according to the inclusion criteria of the J-POP registry. Subsequently, 1,382 patients who were declared dead in the emergency room despite advanced life support, 26 patients for whom data regarding demographic factors were lacking, and 18 patients with Hb levels of <7.0 g/dl were excluded, and the data of the remaining 495 patients were analyzed (Figure 1). The demographic data are presented in Table 1. Of the 495 patients enrolled, 75 (15.2%) had a good outcome at 90 days after cardiac arrest (CPC 1 = 57 and CPC 2 = 18). Among the remaining 420 patients (84.8%) with a poor outcome, 8, 26, and 386 were diagnosed as CPC 3, CPC 4, and CPC 5, respectively. The 90-day mortality rate of all the enrolled patients was 78.0%.

**Table 1:** **Demographic factors and baseline characteristics of the enrolled patients**

**Variable**	**All patients (** ***n*** **= 495)**	**Good neurologic Outcome (** ***n*** **= 75)**	**Poor neurologic Outcome (** ***n*** **= 420)**
rSO_2_, median (25^th^, 75^th^ percentiles), % ^b^	19 (15, 47)	61 (43, 65)	15 (15, 36)
Hemoglobin, mean (SD), g/dl ^b^	12.1 (2.3)	13.9 (2.1)	11.7 (2.2)
Oxy-Hb, median (25^th^, 75^th^ percentiles) ^b^	2.3 (1.7, 5.2)	8.3 (5.5, 9.7)	2.2 (1.7, 3.9)
Age, mean (SD), years ^b^	68 (16)	59 (14)	69 (15)
Male sex, *n* (%)^a^	330 (66.7)	58 (77.3)	272 (64.8)
Location of cardiac arrest, *n* (%)^b^			
Home	261 (52.7)	27 (36.0)	234 (55.7)
Nursing home/Assisted living	48 (9.7)	4 (5.3)	44 (10.5)
Public building	43 (8.7)	14 (18.7)	29 (6.9)
Street	47 (9.5)	12 (16.0)	35 (8.3)
Others	96 (19.4)	18 (24.0)	78 (18.6)
Bystander–witness status, *n* (%)^b^	344 (69.5)	67 (89.3)	277 (66.0)
Type of bystander witness status, *n* (%)^b^			
No witness	151 (30.5)	8 (10.7)	143 (34.0)
Family member	177 (35.8)	27 (36.0)	150 (35.7)
EMS	45 (9.1)	8 (10.7)	37 (8.8)
Others	122 (24.6)	32 (42.7)	90 (21.4)
CPR initiated by bystander, *n* (%)^b^	199 (40.2)	47 (62.7)	152 (36.2)
Presumed cardiac etiology, *n* (%)^b^	291 (58.8)	64 (85.3)	227 (54.0)
Initial shockable rhythm, *n* (%)^b^	121 (24.4)	43 (57.3)	78 (18.6)
Pre-hospital procedures by EMS personnel, *n* (%)			
Advanced airway device^a^	278 (56.2)	25 (33.3)	253 (60.2)
Use of epinephrine^a^	155 (31.3)	13 (17.3)	142 (33.8)
Defibrillation^b^	146 (29.5)	50 (66.7)	96 (22.9)
Time from EMS call to hospital arrival, mean (SD), min^b^	34.7 (14.5)	30.3 (16.2)	35.5 (14.0)
Achievement of ROSC prior to hospital arrival, *n* (%)^b^	119 (24.0)	55 (73.3)	64 (15.2)
Treatment given after hospital arrival, *n* (%)			
Coronary angiography^b^	137 (27.7)	53 (70.7)	84 (20.0)
Primary percutaneous coronary intervention^b^	57 (11.5)	20 (26.7)	37 (8.8)
Therapeutic hypothermia^b^	187 (37.8)	56 (74.7)	131 (31.2)

EMS, emergency medical service; Oxy-Hb, oxyhemoglobin; ROSC, return of spontaneous circulation. ^a^ *P* < 0.05, ^b^ *P* < 0.001 between a good-outcome group and a poor-outcome group.

### Levels of rSO_2_, Hb, and estimated cerebral Oxy-Hb at hospital arrival

**Figure 2:**
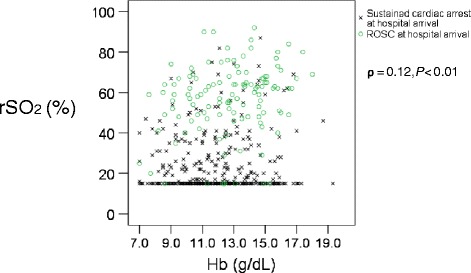
**The relation between rSO**_**2**_ **and hemoglobin during resuscitation at hospital arrival.** Oxy-Hb, estimated oxy-hemoglobin; rSO_2_, regional cerebral oxygen saturation; ROSC, return of spontaneous circulation.

The 495 patients had a median rSO_2_ value of 19% (15% to 47%) and mean Hb levels of 12.1 ± 2.3 g/dl (Table 1). The relation between the rSO_2_ value and Hb levels at hospital arrival is shown in Figure 2. Including the patients who continued to receive CPR and arrived at hospital immediately after ROSC, rSO_2_ was not correlated with Hb levels (Spearman rank correlation coefficient: ρ = 0.12; *P* < 0.01). For all patients, the oxy-Hb level ranged from 1.0 to 13.9, with a mean of 3.8 ± 2.8 and a median of 2.3 (1.7 to 5.2).

### Estimated Oxy-Hb levels and arterial pH at hospital arrival

**Figure 3:**
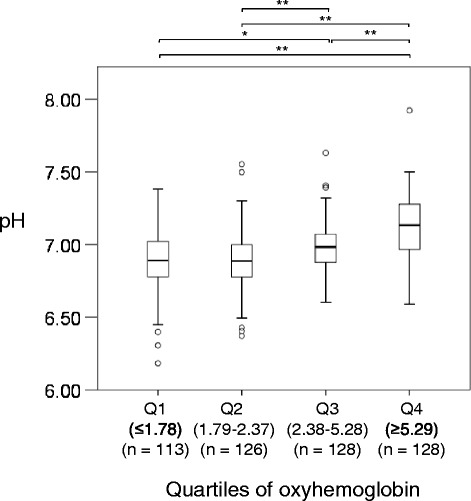
**The relation between estimated oxyhemoglobin levels and arterial pH at hospital arrival.** Hb, hemoglobin; Oxy-Hb, estimated oxy-hemoglobin; rSO_2_, regional cerebral oxygen saturation; *P < 0.01, **P < 0.001.

The relation between the estimated oxy-Hb level and arterial pH at hospital arrival is shown in Figure 3. The arterial pH was significantly higher with higher oxy-Hb quartiles [pH, 1^st^ quartile of oxy-Hb: 6.88 (6.77, 7.02); 2^nd^ quartile: 6.89 (6.77, 7.00); 3^rd^ quartile: 6.97 (6.87, 7.05); and 4^th^ quartile: 7.13 (6.95, 7.27), *P* < 0.001].

### Estimated Oxy-Hb levels as predictors of neurologic outcomes in patients with PCAS

**Figure 4:**
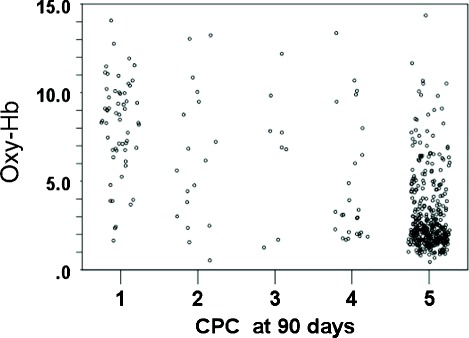
**The relation between estimated oxy-Hb at hospital arrival and cerebral performance category.** CPC, cerebral performance category; Oxy-Hb, oxyhemoglobin.

Univariate analyses revealed that the following potential predictors were significantly associated with the primary outcome: Hb levels, rSO_2_, oxy-Hb levels, age, sex, bystander–witness status, bystander CPR, presumed cardiac etiology, initial shockable rhythm, advanced airway management by EMS personnel, epinephrine use, defibrillation by EMS personnel, time interval from EMS call to hospital arrival, achievement of ROSC before hospital arrival, administration of coronary angiography, primary percutaneous coronary intervention, and therapeutic hypothermia (Table 1). A significant association was noted between oxy-Hb levels and the primary outcome (unit odds ratio (OR): 1.60; 95% CI, 1.45 to 1.76; *P* < 0.001) after regression analysis. The association between oxy-Hb levels and good neurologic outcomes at 90 days is shown in Table 2 and Figure 4. Multivariate analysis was adjusted for oxy-Hb, age, sex, bystander–witness status, presumed cardiac etiology, bystander CPR, initial shockable rhythm, achievement of ROSC before arrival at the hospital, time interval from EMS call to hospital arrival, and administration of therapeutic hypothermia. The results revealed that the oxy-Hb level was a significant predictor of good neurologic outcomes (adjusted unit OR, 1.27; 95% CI, 1.11 to 1.46; *P* < 0.001), such that a 1-unit increase in oxy-Hb levels was associated with an approximate 30% increase in the odds of a good neurologic outcome. Even in the subgroup of patients without ROSC at hospital arrival (*n* = 376; 20 patients with good neurologic outcomes), the oxy-Hb level was significantly associated with an increase in good neurologic outcomes (OR, 1.66; 95% CI, 1.30 to 2.12; multivariate logistic regression using backward elimination; *P* < 0.001).

**Table 2:** **Multiple logistic regression model using backward-elimination method, with good neurologic outcome at 90 days as the dependent variable**

**Variable**	**OR**	**95%CI**
Oxy-Hb (per 1 increase)	1.27	1.11 to 1.46
ROSC before hospital arrival	6.78	2.66 to 17.28
Presumed cardiac etiology	2.93	1.14 to 7.56
Initial shockable rhythm	2.53	1.10 to 5.79
Bystander–witness status	2.29	1.14 to 4.59
Age	0.98	0.95 to 0.99
Therapeutic hypothermia	1.67	0.75 to 3.70
Time interval from EMS call to hospital arrival	0.98	0.95 to 1.00

Selected variables are a predefined set of potential confounders including age, sex, initial shockable rhythm, bystander-witness status, CPR initiated by bystander, ROSC before hospital arrival, presumed cardiac etiology,

time interval from EMS call to hospital arrival, therapeutic hypothermia, and oxy-Hb. The Hosmer-Lemeshow tests.

were used to assess the goodness of fit of the model (*P* > 0.5).

EMS, emergency medical service; OR, odds ratio; Oxy-Hb, oxyhemoglobin; ROSC, return of spontaneous circulation.

**Figure 5:**
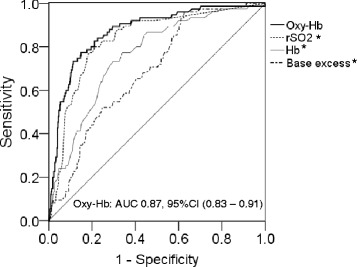
**AUC of each potential indicator for predicting neurologic outcome at 90 days.** The area under the receiver operating characteristic curve (AUC) of each potential indicator to predict good neurologic outcome at 90-day hospital admission in patients with post–cardiac arrest syndrome. *P < 0.001 versus AUC of oxyhemoglobin.

We also evaluated the sensitivity and specificity of different oxy-Hb, rSO_2_, Hb, and base excess cut-off values among the entire cohort. ROC analysis revealed cut-offs providing optimal sensitivity and specificity to predict good neurologic outcomes at 90 days, which are summarized in Table 3. In this cohort, oxy-Hb was the most reliable neurologic prognostic index, with significant advantages over rSO_2_, Hb, and base excess (*P* < 0.001, Table 3 and Figure 5).

**Table 3:** **Optimal cut-off value of oxy-Hb, rSO**_**2**_ **, Hb, and base excess at hospital arrival for predicting a good neurologic outcome at 90 days**

**Variables**	**Optimal cut-off**	**AUC (95% CIs)**	**Sensitivity (95% CIs)**	**Specificity (95% CIs)**	**PPV**	**NPV**	***P*** **value (** ***versus*** **Oxy-Hb)**
Oxy-Hb	5.5	0.87 (0.83 – 0.91)	77.3% (72.4 – 82.1)	85.0% (83.2 – 86.7)	47.9%	95.4%	N/A
rSO_2_	40%	0.83 (0.78 – 0.88)	80.0% (75.3 – 84.6)	78.6% (76.5 – 80.6)	40.0%	95.6%	< 0.001
Hb	13.0 g/dl	0.77 (0.70 – 0.81)	73.3% (68.1 – 78.4)	70.2% (69.7 – 72.4)	30.5%	93.6%	< 0.001
Base excess	−18.7 m*M*	0.68 (0.63 – 0.74)	96.0% (93.7 – 98.2)	37.4% (35.0 – 39.7)	21.4%	98.1%	< 0.001

AUC, area under the curve; CI, confidence interval; Hb, hemoglobin, NPV, negative predictive value; Oxy-Hb, oxyhemoglobin; PPV, positive predictive value; rSO_2_, regional cerebral oxygen saturation.

**Figure 6:**
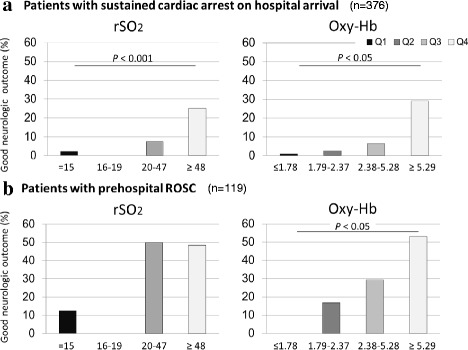
**The association between rSO**_**2**_ **and estimated cerebral oxy-hemoglobin and frequency of good neurologic outcomes.** Study participants were divided into four groups by oxy-Hb and rSO_2_ quartiles (Q1, *n* = 126; Q2, *n* = 122; Q3, *n* = 124; Q4, *n* = 123 for oxy-Hb: Q1, *n* = 221; Q2, *n* = 28; Q3, *n* = 125; Q4, *n* = 121 for rSO_2_). **(a)** Subgroup of patients with sustained cardiac arrest at hospital arrival (*n* = 376). **(b)** Subgroup of patients with ROSC at hospital arrival (*n* = 119). Oxy-Hb, estimated oxyhemoglobin; rSO_2_, regional cerebral oxygen saturation; ROSC, return of spontaneous circulation.

Finally, we classified patients into four groups by oxy-Hb and rSO_2_ quartiles to assess the relation between these quartiles and the primary outcomes in the subgroups of patients with and without ROSC at hospital arrival. In the subgroup of patients with sustained cardiac arrest at hospital arrival, a significant trend was found for a good neurologic outcome with increasing oxy-Hb levels and rSO_2_ (Figure 6a). Of the 119 comatose patients with ROSC at hospital arrival, 55 (46.2%) had a good outcome at 90 days after cardiac arrest. Even in this subgroup, the trend was significant for good neurologic outcomes across increasing oxy-Hb quartiles (1^st^ quartile, 0; 2^nd^ quartile, 16.7%; 3^rd^ quartile, 29.4%; 4^th^ quartile, 53.3%; Cochran–Armitage trend test, *P* < 0.05) but not across rSO_2_ quartiles (12.5%, 0; 50.0%, and 48.4%, respectively; *P* = 0.14) (Figure 6b). In this subgroup, AUC to predict good neurologic outcomes at 90 days for oxy-Hb (AUC, 0.67; 95% CI, 0.58 to 0.77; *P* = 0.001) was superior to that for rSO_2_ (AUC, 0.56; 95% CI, 0.45 to 0.66; *P* > 0.05).

## Discussion

In this study, the estimated cerebral oxy-Hb level on hospital arrival was a valid indicator of 90-day neurologic outcome in patients who successfully achieved ROSC from OHCA. The estimated oxy-Hb level was easily and immediately obtained on hospital arrival. We verified the hypothesis that oxy-Hb is associated with neuroprotection in patients with PCAS on the basis of the theoretical assumption that the product of Hb and rSO_2_ may reflect cerebral tissue oxy-Hb levels. Multivariate analyses revealed that the oxy-Hb value was a significant predictor of good neurologic outcomes in the entire cohort. AUC was significantly greater for oxy-Hb than for rSO_2_, Hb, and base excess, which were independently shown to be associated with neurologic outcomes [23,24]. Moreover, our results revealed that the oxy-Hb level determined in the immediate post-ROSC period could reflect the severity of neuroprotection, even in a subgroup of patients with ROSC before hospital arrival.

In a previous prospective multicenter cohort study, it was reported that, on the basis of specificity, positive predictive value, and AUC, rSO_2_ of >42% at hospital arrival was an excellent neurologic prognostic index in OHCA (including patients who received ongoing CPR) and that rSO_2_ also had advantages over base excess [25]. In the present subgroup of patients who successfully achieved ROSC from OHCA, both rSO_2_ and oxy-Hb levels were strong predictors of neurologic outcomes, which was consistent with the results of the previous study [25]. However, our study revealed that it was difficult to predict neurologic outcomes with rSO_2_ alone if patients had already achieved ROSC before hospital arrival (Figure 6a). This is probably because cerebral oximetry typically increases after ROSC [12]. Although it has been shown that hyperoxia is associated with adverse outcomes after resuscitation [26], supporting the concept that supranormal arterial oxygen tension over the first 24-hour period in the intensive care unit (ICU) could be harmful to humans [26], our data suggest that increased circulating oxy-Hb in brain tissue may be crucial for neuroprotection during CPR.

A meta-analysis by Sasson *et al*. [19] indicated that ROSC in the field is the most powerful factor associated with survival from OHCA, which was consistent with our results (Table 2). Therefore, surrogates of neuroprotection are required in patients with pre-hospital ROSC. Our results indicated that increasing oxy-Hb levels reflected progressively superior neurologic outcomes, even in patients in the immediate post-ROSC period (Figure 6b).

Tissue metabolism and cerebral oxy-Hb depend on a balance between oxygen consumption, blood Hb levels, blood oxygenation, and tissue perfusion. Insufficient cerebral perfusion and subsequently reduced cerebral NIRS and oxy-Hb readings during CPR or after ROSC are most likely caused by low cardiac output. However, the differential diagnosis for the low cerebral NIRS readings also includes intracranial hematomas [27], low arterial oxygen saturation [28], and isolated cerebral circulatory arrest [29,30]. Although blood Hb levels theoretically play a crucial role in maintaining tissue metabolism, this has not yet been sufficiently studied during CPR. To resolve the tissue oxygen debt during cardiac arrest, blood circulation must be secured by high-quality and less-interrupted chest compressions [7]. In our study, a significant relation was found between oxy-Hb levels and arterial pH, suggesting that increasing oxy-Hb levels during CPR may attenuate metabolic acidosis by appropriate chest compression. Because rSO_2_ may reflect the balance between oxygen delivery and regional (frontal) cerebral metabolism, the product of blood Hb levels and cerebral oxygen saturation could parallel oxygen delivery during resuscitation. Therefore, rSO_2_ measured during CPR attempts could indicate whether the ongoing attempt is likely to sustain adequate oxygen delivery to cerebral tissues [31].

The clinical experience of cardiac-arrest resuscitation demonstrates that circulatory recovery does not always coincide with cerebral recovery. The high morbidity of neurologic dysfunction in patients with ROSC after OHCA has led to several clinical studies attempting to optimize neurologic outcomes through therapeutic efforts that include targeted temperature management or administration of neuroprotective medications [32-34]. Our data are consistent with this clinical experience: although a standard CPR method may deliver adequate coronary perfusion to reverse cardiac arrest, it may deliver inadequate oxy-Hb to the central nervous system and contribute to cellular damage in cerebral tissue.

The ability to predict outcomes early is an important aspect of postresuscitation care in patients with PCAS. A multimodal prediction approach has been evaluated for outcome prognostication after cardiac arrest and therapeutic hypothermia [35]. This incorporates neurologic examination [36,37], electroencephalography [37,38], somatosensory evoked potentials [39,40], neuron-specific enolase [41,42], and magnetic resonance imaging [43-45]. Multimodal scores that combine both clinical and laboratory variables have also been developed for outcome prediction. The OHCA score presented by Adrie *et al*. [46] predicts good neurologic outcomes for patients with AUC of 0.79 in a heterogeneous population of successfully resuscitated adult patients with OHCA. The advantage of prognostication using oxy-Hb is that prognostic assessment can be readily performed at hospital arrival. Moreover, modern multimodal severity-scale scores predict survival with an AUC of approximately ≥0.80 after further confounders are included [47,48], whereas oxy-Hb at hospital arrival has an AUC of 0.87. Thus, oxy-Hb has real potential as an indicator of neuroprotection in patients with PCAS.

Recently, Nielsen *et al.* [49] described that therapeutic hypothermia at a target temperature of 33°C conferred no additional benefit compared with that at a targeted temperature of 36°C. One of several explanations for this absence of benefit is that illness severity varies greatly, and appropriate subgroups of patients may benefit from induced hypothermia. In particular, when the degree or duration of hypothermia must be adjusted to match injury severity, the benefits to a subgroup may be masked if appropriate subgroups are not defined [50]. Our results provide the possibility for estimated cerebral oxy-Hb levels to define subgroups that may benefit from individual therapies and to clarify how to adjust temperature targets to particular severities.

This study had several limitations. First, blind monitoring of rSO_2_ was not conducted because it requires real-time visual confirmation during CPR efforts; this did not affect day-to-day patient care. Second, because of the overall mortality of 78.0%, the small cohort, and the fact that only 75 patients had good neurologic outcomes, future research with more patients is essential to validate our findings. Third, patients with significant anemia at the time of enrollment (Hb < 7.0 g/dl) were excluded from the present study; thus, our data are applicable only to patients with modest Hb reductions. Fourth, we were unable to control for blood bilirubin concentration in this study, and given that bilirubin is known to influence NIRS [51,52], it may have influenced the readings. Finally, rSO_2_ was measured only at a single measurement point in this study. We recognized the importance of understanding the impact of time on changes in rSO_2_ for the prognostication of neurologic outcomes. Thus, we are presently collecting rSO_2_ data regularly during admission to the ICU. Furthermore, neither the relation between rSO_2_ and Hb levels over time nor the clinical effect of red blood cell transfusion on outcomes is clear. Thus, although the results of this study are promising, they remain inconclusive on whether to continue or withdraw care for patients with PCAS.

## Conclusions

In summary, our data provide evidence that the estimated oxy-Hb level may help to predict favorable 90-day neurologic outcomes in patients with PCAS. Furthermore, our study demonstrated that the oxy-Hb level determined immediately on hospital arrival was significantly higher in patients with good outcomes (AUC = 0.87), making it a simple and effective indicator of neuroprotection in patients with PCAS. Thus, the estimated oxy-Hb level may be included as part of a multimodal package for prognostication after cardiac arrest and warrants larger-scale studies.

## Key messages

- The ability to predict outcomes early is an important aspect of postresuscitation care in patients with PCAS.
- Cerebral oximetry with NIRS has been developed as a noninvasive technology that may be used for monitoring cerebral oxygen saturation during cardiac arrest.
- Oxy-Hb levels and cardiac output are essential determinants of oxygen delivery during ongoing CPR attempts.
- The estimated oxy-Hb level obtained on hospital arrival may help to predict favorable 90-day neurologic outcomes in patients who are successfully resuscitated after OHCA.

## Abbreviations

AUC: Area under the receiver operating characteristic curve
CI: confidence interval
CPC: cerebral performance category
CPR: cardiopulmonary resuscitation
EMS: emergency medical service
ICU: intensive care unit
J-POP: Japan Prediction of neurological Outcomes in patients with Post cardiac arrest
NIRS: near-infrared spectroscopy
NPV: negative predictive value
OHCA: out-of-hospital cardiac arrest
OR: odds ratio
oxy-Hb: oxyhemoglobin
PCAS: post–cardiac arrest syndrome
PPV: positive predictive value
ROC: receiver operating characteristic
ROSC: return of spontaneous circulation
rSO_2_: regional cerebral oxygen saturation
SD: standard deviation
